# Maintenance of homeostasis by TLR4 ligands

**DOI:** 10.3389/fimmu.2024.1286270

**Published:** 2024-04-23

**Authors:** Masataka Oda, Hirofumi Yamamoto, Takashige Kawakami

**Affiliations:** ^1^ Control of Innate Immunity, Technology Research Association, Takamatsu, Kagawa, Japan; ^2^ Faculty of Pharmaceutical Sciences, Tokushima Bunri University, Tokushima, Japan

**Keywords:** TLR4 ligands, macrophage, macrophage network, innate immunity, self-healing ability

## Abstract

Immunotherapy is renowned for its capacity to elicit anti-infective and anti-cancer effects by harnessing immune responses to microbial components and bolstering innate healing mechanisms through a cascade of immunological reactions. Specifically, mammalian Toll-like receptors (TLRs) have been identified as key receptors responsible for detecting microbial components. The discovery of these mammalian Toll-like receptors has clarified antigen recognition by the innate immune system. It has furnished a molecular foundation for comprehending the interplay between innate immunity and its anti-tumor or anti-infective capabilities. Moreover, accumulating evidence highlights the crucial role of TLRs in maintaining tissue homeostasis. It has also become evident that TLR-expressing macrophages play a central role in immunity by participating in the clearance of foreign substances, tissue repair, and the establishment of new tissue. This macrophage network, centered on macrophages, significantly contributes to innate healing. This review will primarily delve into innate immunity, specifically focusing on substances targeting TLR4.

## 1 Introduction

The immune system is essential to protect the host from invading pathogens and abnormal cells such as cancer cells and is primarily classified as innate and acquired immunity. Innate immunity is an immediate general defense function that we are born with, while acquired immunity has the ability to learn, respond specifically, and memorize responses to specific pathogens. This memory system allows the host organism to respond more rapidly and effectively when exposed to the same or related antigens. In recent years, research has focused on the innate immune memory system, particularly macrophages. This research has not only shed new light on host defense mechanisms but also has significant implications for vaccination and allergy strategies (1–3).

The correlation between infection and remission of malignancy was initially observed in the 18th century (1, 2). Dr. William Corey, an American surgeon of the 18th century, is recognized as a trailblazer in this domain. In the 1890s, he demonstrated the regression of cancer in a patient who developed a bacterial infection following surgery for sarcoma. Intrigued by this observation, Dr. Coley administered live cultures of streptococci to induce erysipelas in cancer patients and assessed their response. He found that the antitumor effect was reliant on the bacterial toxin. Eventually, he combined toxins from a gram-positive bacterium (*Streptococcus pyogenes)* with those from a gram-negative bacterium (Serratia marcescens), naming it “Coley toxin” (also known as “mixed bacterial vaccine”) (3). Dr. Cawley and his daughter, Dr. Helen Cawley Notes, continued the pioneering efforts in treating cancer based on immune system function.

It has been demonstrated that innate immune responses to microorganisms can induce anti-tumor and anti-infective effects akin to those of Coley toxins, and a diverse array of immune-inducing bacterial-derived and other substances have been identified as “biological response modifiers (BRMs).”

### 1.1 Toll-like receptors

Cells of the innate immune system possess the ability to detect infectious agents through receptors that recognize characteristic components of pathogenic microorganisms. These components, known as pathogen-associated molecular patterns (PAMPs), exhibit high conservation and are encoded in the germ line, making them highly conserved across different species. Toll, initially recognized for its involvement in establishing the dorsal-ventral polarity in the Drosophila embryo (4), has also been found to participate in the innate immune response to fungi (5). In 1997, a mammalian homolog of Drosophila Toll was cloned (6), and to date, ten human molecules, known as Toll-like receptors (TLRs), have been confirmed (7, 8).

The discovery of TLRs in mammals has the potential to offer insights into the molecular basis of early host defense processes against microbial infections. Moreover, accumulating evidence suggests that TLRs play a diverse role in various biological processes. Like other pattern recognition receptors (PRRs), TLRs exhibit a repeated leucine-rich motif in their extracellular domain and a conserved intracellular motif known as the Toll/interleukin-1 receptor (TIR) domain, which initiates signal transduction. TLRs are type I transmembrane proteins.

The initial stages of Toll-like receptor (TLR) signaling involve the mediation of adaptor molecules, namely Myeloid differentiation factor 88 (MyD88), Toll-like receptor-associated activator of interferon (TRIF), MyD88 adaptor-like protein (MAL/TIRAP), Toll receptor-associated molecule (TRAM), and other adapter molecules, which interact with downstream components like NF-κB, JNK/p38 kinase, and interferon regulatory factors (IRF3, IRF5, and IRF7). Ultimately, TLR signaling triggers the expression of diverse transcripts, including cytokines and genes that are induced by interferon (IFN).

### 1.2 Role of TLRs in tissue homeostasis

The Toll-like receptors (TLRs) play a significant role in maintaining tissue homeostasis, beyond their primary function in host defense (9, 10). They are involved in recognizing various endogenous ligands released from dead cells in injured or infected tissues, termed damage-associated molecular patterns (DAMPs). These ligands include uric acid crystals, surfactant protein A, fibronectin (an extracellular matrix product), heparan sulfate, biglycan, fibrinogen, and hyaluronan oligosaccharides, as well as hyaluronan degradation products (11–19), which trigger TLR activation.

Wound healing is a complex process through which injured organs undergo repair (20). TLR activation can contribute to tissue damage correction, either positively or negatively, by recruiting inducible cells that release cytotoxic mediators or by triggering cytoprotective signals (21, 22). TLRs exhibit cytoprotective properties and prevent tissue injury under stress conditions in the lung and intestine. For instance, in bleomycin-induced lung injury, interactions between hyaluronic acid and TLR2/TLR4 signal the initiation of an inflammatory response, maintaining epithelial cell integrity, and promoting recovery from acute lung injury (19). In a model of intestinal injury induced by dextran sodium sulfate, TLR4 and MyD88 signaling are required for optimal proliferation and protection from apoptosis of the injured intestine. Additionally, activation of TLRs by commensal microflora is crucial for protecting against intestinal injury and associated mortality (23, 24). However, TLR4 has been shown to promote injury in ischemia-reperfusion experiments involving the liver, kidney, brain, and heart using TLR4 mutant or TLR4-deficient mice (25–28). In the central nervous system, TLRs coordinate protective responses to axonal injury and crush in the brain and spinal cord (29–31).

The involvement of TLRs in tissue and organ regeneration is evident in cases like the regeneration of the liver after partial hepatectomy. The regenerative response involves multiple biological functions, including cell proliferation, angiogenesis, extracellular matrix reconstruction, and epithelialization (32). TLRs also regulate compensatory proliferation of parenchymal cells after injury (24, 32, 33), induce cyclooxygenase, chemokines, vascular endothelial growth factor (VEGF), matrix metalloproteinases (23, 24, 34), and activate mesenchymal stem cells (35). Thus, TLRs play a pivotal role throughout the entire process of tissue repair and regeneration, significantly contributing to tissue homeostasis. During evolution, TLRs may have acquired dual roles in tissue homeostasis, involving the regulation of the body’s dynamics and the promotion of regenerative processes.

Recently, immune checkpoint inhibitors have been used in cancer therapy and have caused dynamic phenotypic changes to macrophages, and severe immune-related adverse events have been reported (36–38). This is thought to be due to the fact that checkpoint inhibitors change the polarity of macrophages from M2 to M1 (39–41). Although M1 and M2 macrophages differ in shape and properties, they both possess TLR receptor families, etc., and it has been suggested that their receptor ligands act to modulate the immune response (42, 43). Therefore, when using immune checkpoint inhibitors, it is important to activate macrophages via TLR4 in terms of maintaining homeostasis, i.e., to maintain a balance between M1 and M2 macrophages.

### 1.3 Maintenance of homeostasis by the macrophage network

Upon encountering external pathogens, macrophages activate intracellular signaling pathways through Toll-like receptors (TLRs) to initiate an immune response against the invading pathogen. Macrophages and dendritic cells, which are antigen-presenting cells, are known to express particularly high levels of TLRs (44, 45). While the precise origin of macrophages remains uncertain, phylogenetics suggest that they may have originated from protozoa with active phagocytic abilities, such as amoebae. Nevertheless, since phagocytic cells are present in all animals, from unicellular protists like amoebas to mammals, macrophages in humans likely play a critical role in maintaining homeostasis. Thus, stimulating TLRs expressed on macrophages and activating the network, which centers around macrophages, could significantly contribute to disease prevention and treatment and, consequently, promote homeostasis (46).

Recent research has identified at least two distinct tissue-endemic stromal macrophages in the steady-state lung. These macrophages exhibit unique transcriptional profiles and are spatially localized in the interstitium of bronchovascular bundles rather than within alveolar walls (47). In fact, it is now understood that most tissues harbor multiple macrophage populations localized to different microanatomical regions (47–49). Each of these populations differs in their developmental mode, replacement rate involving monocyte-derived cells, and self-renewal capacity. Moreover, each population may play a specific role in maintaining tissue homeostasis, responding to injury, and participating in tissue repair processes (43, 50–52).

Cells with TLR4 are abundant in the innate immune system and include macrophages and mucosal epithelial cells. Unlike all other cells, macrophages are systemically distributed and account for about half of the immune cells by weight (53). Macrophages are known to be migratory and active in migrating to lesions, processing not only invading foreign substances (bacteria, viruses, etc.) but also dead and senescent cells, degenerated proteins, oxidized lipids, AGEs, and other unwanted substances generated by the body, and are responsible for repair and regeneration of damaged tissue.

Macrophages (phagocytes) are ubiquitous in multicellular animals, but have been shown to play an important role in individual health even in those without a nervous system in early stages of evolution (54). In other words, in multicellular animals, there must have been a mechanism for maintaining individual integrity that predates the emergence of the nervous system. Based on our previous findings, we can predict the existence of a mechanism that maintains individual homeostasis through signal transduction between migrating macrophages. This macrophage-mediated signal transduction system has been proposed as the macrophage network hypothesis (46). Fujii et al. In a mouse model of pressure overload on the heart, kidney tissue macrophages were found to secrete M- CSF2 secretion and reported a network that activates cardiac tissue macrophages to increase cardiomyocyte (55). It is also speculated that the TLR4-mediated activation system acts as the first signal in macrophage network. In fact, Mizobuchi et al. have shown that orally administered LPS (lipopolysaccharide) induces membrane-bound CSF1 in peripheral blood leukocytes, which stimulates CSF1 receptors in brain microglia, and neuroprotective and anti-inflammatory effects have been shown in brain diabetes-induced mice (56). These reports suggest that systemic macrophages interact in mammals and that TLR4 signaling plays a role in their regulation.

### 1.4 Exogenous immunostimulants

#### 1.4.1 LPS

LPS is a glycolipid present in the extracellular membrane of Gram-negative bacteria and is a known ligand that activates TLR4 at trace amounts of only a few pg/ml (57). LPS is released from Gram-negative bacteria as exosomes into the environment from survival as outer membrane vesicles, and dead Gram-negative bacteria readily release LPS (58, 59). Therefore, LPS is present wherever symbiotic Gram-negative bacteria are present on the mucosa of animals’ skin, oral cavity, airways, and intestinal tract, and it is thought that TLR4-mediated exchange is used as an information molecule from the symbiotic bacteria to and from the animal. LPS is known to induce antimicrobial peptides from Paneth cells in the small intestine (60), which contribute to the stabilization of intestinal bacteria, and to transmit information from tissue macrophages in the large intestine to neurons, which induce intestinal peristalsis (61).

Studies utilizing LPS have unveiled the importance of TLR ligands administered orally or transdermally in activating the macrophage network without inducing inflammatory reactions (56, 60, 62, 63). However, it is believed that oral and transdermal LPS administration activates the systemic macrophage network through mucosal macrophages. The involvement of the TNF superfamily and membrane-bound colony-stimulating factor 1 (mCSF1) in this macrophage network is suspected, although the specific mechanisms remain unclear.

Notably, animal experiments involving oral LPS administration have demonstrated its associations with various conditions, including cancer, Alzheimer’s disease, ulcers, viral infections, toxoplasma infections, allergies, hyperlipidemia, hypertension, diabetes, and hair growth. Clinical trials in humans have further revealed effects on cancer, atopy, diabetes, capillary growth, wound healing, and developmental disorders (64–70). Notably, recent findings suggest that oral LPS administration enhances the foreign body processing function of microglia, which are brain macrophages, providing a preventive effect against Alzheimer’s disease (56). These effects induced by oral LPS administration are likely a result of the indirect influence of cytokines, including membrane-bound types triggered by stimulation of innate immune sensor cells in the mucosal epithelium. These cytokines subsequently act on tissue macrophages in the juxtacrine and paracrine manner, contributing to the macrophage network.

Furthermore, sublingual administration of LPS has been shown to significantly enhance the efficacy of influenza vaccines and reduce mortality (71, 72). As a result, sublingual LPS administration holds promise in preventing and treating emerging infectious diseases, including those caused by novel coronavirus infections expected to arise in the future.

#### 1.4.2 Fucoidan

Fucoidan, a sulfated polysaccharide abundant in the cell walls of brown algae and marine organisms, possesses a highly complex chemical composition that varies depending on the algal source, geographical location, and extraction process (1). The structural backbone of fucoidan consists of fucopyranose residues with repeated α-(1→3) bonds or L-fucopyranose residues with alternating α-(1→3) and α-(1→4) linkages. These fucosyl groups may be mono- or di-substituted with sulfate or acetate groups at C-2, C-4, and occasionally at C-3 (73, 74). Additionally, fucoidan structures may contain a variety of other monosaccharides (mannose-type), such as mannose, galactose, arabinose, xylose, glucose, uronic acid, and proteins, in addition to the fucosyl main chain (75).

Absorption studies with fucoidan (737 kDa) were conducted in rats. After administration, the concentration of fucoidan in the serum reached a maximum and absorbed fucoidan accumulated. Absorbed fucoidan accumulated in the kidneys. The accumulation of fucoidan in organs has also been demonstrated by the absorption of fucoidan in rats (76). In addition, observations of healthy volunteers who ingested or received fucoidan orally reported that some of the fucoidan was absorbed by endocytosis and was detected in serum and urine (77).

In recent years, fucoidan derived from algae has been the subject of intensive research due to its diverse biological activities and therapeutic potential. Fucoidan has shown interactions with TLR2 and TLR4 (78, 79), and various pharmacological effects have been reported, including antitumor, immunomodulatory, antiviral, antibacterial, antidiabetic, renoprotective, antioxidant, anti-inflammatory, and anticoagulant effects (80–82). Moreover, fucoidan has been investigated for its application in improving various pathological conditions, such as diabetes, hepatic lipidosis (fatty liver), liver damage, renal ischemia, abnormal blood coagulability, stem cell therapy, gastric ulcer, gout, bacterial and viral infections, and snakebite (73, 78, 79, 81–83). The wide range of biological activities makes fucoidan a potential candidate for immune response modulation, antibacterial and antiviral agents (81).

Developing standardized fucoidan supplements is a complex process, as factors such as raw materials, species, molecular weight, composition, structure, and route of administration significantly impact the efficacy of the compounds. Additionally, most of the reported activities are based on in vitro experiments or in vivo evaluations using laboratory animals. Care should be taken as different animal models may produce varying effects when evaluated in different contexts.

Despite the large number of studies on fucoidan, few clinical trials have been planned and conducted. In most cases, different cell lines and animal models have been used to study different types of fucoidan. This makes it difficult to determine the general mechanism of action for a particular type of fucoidan. There is also little information on the absorption, distribution, and excretion of fucoidan. Although the biological activities exhibited by fucoidan are fascinating, most of these studies have been conducted on relatively crude fucoidans, making it very difficult to determine the structure-activity relationship.

#### 1.4.3 Vizantin

In 1956, Dr. Chisato Maruyama observed that there were few cancer patients in sanatoriums for tuberculosis and leprosy, which led to research on the application of extracts of Mycobacterium tuberculosis for cancer treatment (84). The prepared extract, called “Specific Substance Maruyama (SSM),” was a deproteinized extract primarily composed of lipoarabinomannan, a type of polysaccharide (85). Another example of a *Mycobacterium tuberculosis*-derived biological response modifier (BRM) is BCG-CWS, a cell wall skeletal preparation of Mycobacterium bovis bacillus Calmette-Guerin. BCG-CWS is largely nonpathogenic but retains the immunogenicity of tuberculosis (85). It is a peptidoglycan covalently bound to arabinogalactan and mycolic acid (86). BCG-CWS has been clinically used as a cancer immunotherapy (87, 88) but has not been approved by the Ministry of Health, Labour, and Welfare (MHLW). These biologically derived classical BRMs are considered “natural compounds” and are characterized as crude products containing multiple components, as they have not undergone full purification.

**Figure 1:**
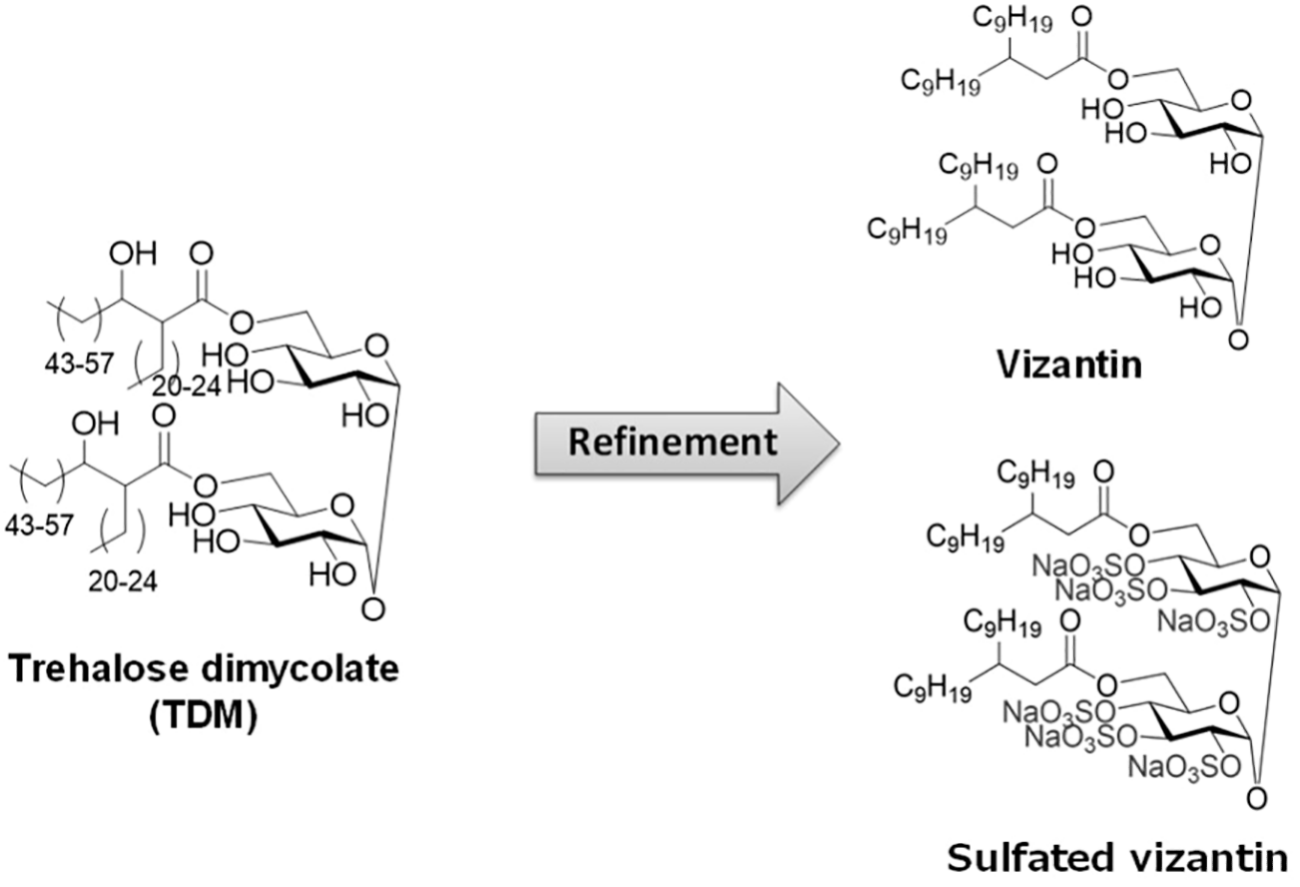
The structure of TDM, vizantin and sulfated vizantin.

Focusing on *Mycobacterium tuberculosis*-derived BRMs, researchers developed Vizantin, a single-component immunostimulant, using trehalose dimycolate (TDM) present on the cell surface layer of *M. tuberculosis* as the lead compound (89). Vizantin is a trehalose diester consisting of two achiral β-branched fatty acids, 2-nonylundecanoic acid, fused to the hydroxyl groups at the 6, 6’ positions of trehalose. Additionally, researchers successfully created sulfated Vizantin by sulfating the hydroxyl group of the trehalose moiety, making it water-soluble (90) (**Figure 1**).

Oral administration of Vizantin to mice has been shown to prevent the settlement of melanoma cells in the lungs, effectively preventing lethality (89). Additionally, intravenous administration of Vizantin or sulfated Vizantin, which has improved water solubility, into the tail vein of mice has been found to prevent lethality caused by multidrug-resistant Pseudomonas aeruginosa infection. The mechanism behind this effect involves sulfated Vizantin acting on macrophages to form extracellular trapping nets (METs), which trap *P. aeruginosa* (91). Further analysis has revealed that sulfated Vizantin does not affect the growth of *P. aeruginosa* but reduces its swimming activity by disrupting the Che system, which is involved in flagellar motility. Moreover, sulfated Vizantin has been shown to inhibit biofilm formation by disrupting the glucosyltransferase production balance of mutans, the bacteria responsible for causing dental caries.

**Figure 2:**
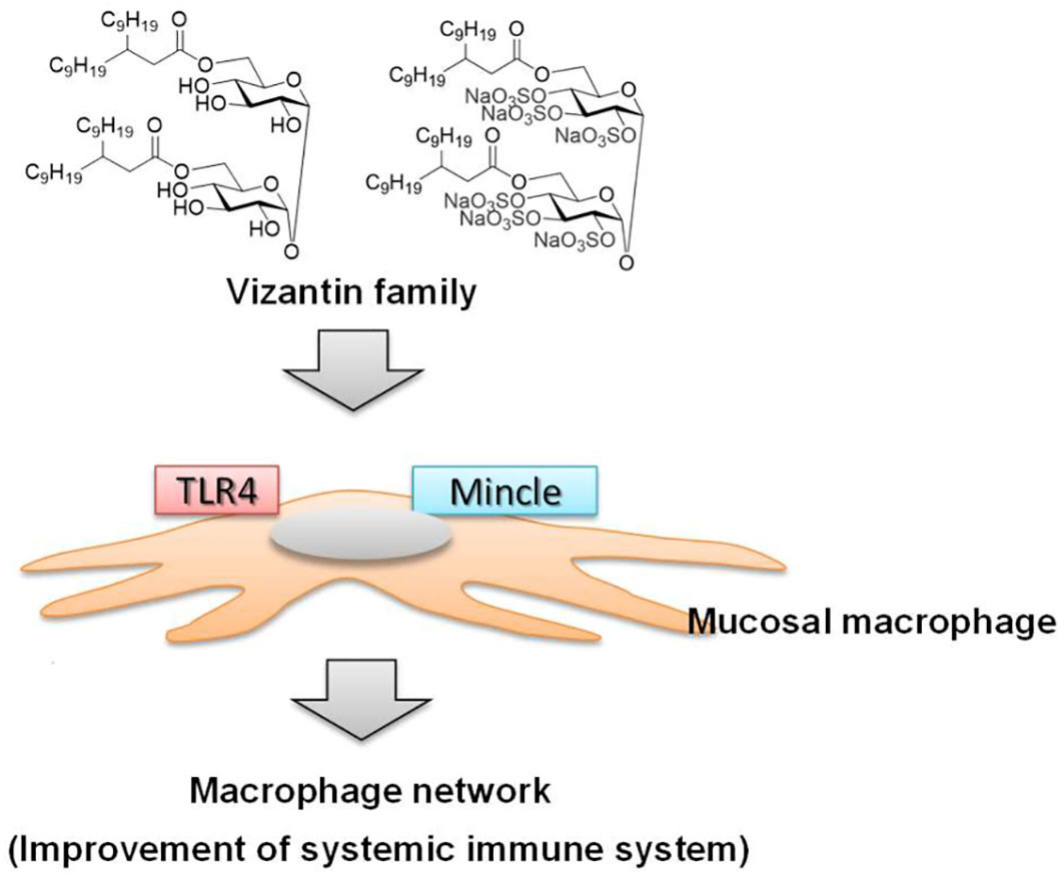
Induction of macrophage network by vizantin family.

Vizantin is also a lead compound of Vizantin, and the glycosylation moiety (trehalose) of TDM, the lead compound of Vizantin, has been demonstrated to act on the Macrophage inducible C-type lectin (Mincle) receptor (92, 93). As Vizantin has a similar binding site to TDM, it is expected that Vizantin also acts on the Mincle receptor. Therefore, the diverse range of responses observed with Vizantin may be attributed to its action on both TLR4/MD-2 and Mincle receptors (**Figure 2**). As a result, it is thought to activate the macrophage network by acting on macrophages in the same way as LPS (**Figure 2**).

### 1.5 Endogenous immunostimulant

#### 1.5.1 HSP70

Heat Shock Protein 70 (HSP70) is a protein that cells induce in response to environmental stimuli, such as stress and heat. Its role is crucial in maintaining protein folding and stability within cells, contributing to cell survival and function. HSP70 also plays a significant role in enhancing the antigen-presenting ability of antigen-presenting cells, like dendritic cells and macrophages. By binding to antigens on these cells, it facilitates the immune system’s recognition of foreign substances and pathogens, leading to an enhanced immune response.

The immune response triggered by HSP70 operates in a CD14-dependent manner through TLR2 and TLR4 receptors (94). This immune response activates cytotoxic T cells (CD8+ T cells), facilitating the elimination of abnormal cells, and regulates the production of inflammation-regulating cytokines and inflammatory cells. The production of cytokines that modulate inflammation and the activation of inflammatory cells are crucial for balancing the immune response. Consequently, HSP70 induced by hyperthermia and other therapies exhibits effects on various diseases, including neurodegenerative disorders like Alzheimer’s and Parkinson’s disease (95), metabolic syndrome (96), cancer (97, 98), and allergic conditions such as asthma (84, 99). These effects are attributed to the activation of the immune system through HSP70 expression, leading to enhanced natural healing capabilities. There are several types of HSPs, and their receptors are being analyzed for their relationship to immune activation, but there are still many unknowns.

## 2 Conclusion

Hippocrates once said, “Man has a hundred great physicians, and the great physician is the power of natural healing.” Today, while appropriate drugs are prescribed for various diseases, they do not cure the diseases themselves. Instead, the human body maintains its health through its own self-healing power. It is important not to overly rely on drugs but rather to strengthen our own immune system and rely on our natural healing abilities. This is especially crucial for dealing with emerging infectious diseases that may not be effectively addressed by individual vaccines or therapeutic agents. When such infectious diseases spread, measures like physical protection such as wearing masks and enhancing individual self-immunity become of utmost importance.

LPS, fucoidan, HSP, and vizantin, which have been discussed here, have been shown to enhance immunity by activating TLRs, especially TLR4, which are pattern recognition receptors. These substances contribute to the prevention and treatment of various diseases. Further research aims to better understand how these substances act on innate immunity, particularly how they influence the function of the macrophage network in maintaining a balanced immune response.

## Author contributions

MO: Conceptualization, Writing – original draft. HY: Writing – review & editing. TK: Writing – review & editing.
